# New Strategies for High Quality of CPR and Post-Resuscitation Care

**DOI:** 10.1155/2022/9818174

**Published:** 2022-02-11

**Authors:** Yan-Ren Lin, Jacek Smereka, Kee-Chong Ng, John M. Ryan

**Affiliations:** ^1^Department of Emergency and Critical Care Medicine, Changhua Christian Hospital, Changhua, Taiwan; ^2^School of Medicine, Kaohsiung Medical University, Kaohsiung, Taiwan; ^3^School of Medicine, Chung Shan Medical University, Taichung, Taiwan; ^4^College of Medicine, National Chung Hsing University, Taichung, Taiwan; ^5^Department of Emergency Medical Service, Wroclaw Medical University, Wroclaw, Poland; ^6^Department of Research Outcomes, Polish Society of Disaster Medicine, Raszyn, Poland; ^7^Department of Emergency Medicine, KK Women's and Children's Hospital, Singapore; ^8^Emergency Department, St Vincent's University Hospital, Elm Park, Dublin, Ireland

Following cardiac arrest, establishing the return of spontaneous circulation (ROSC) and the neurological function of the patient is the key, and the quality of the cardiopulmonary resuscitation (CPR) has a direct effect on the protection of the central and distal vital organs. In addition to traditional chest compression, high-performance cardiopulmonary resuscitation (HPCPR) and extracorporeal cardiopulmonary resuscitation (ECPR) are thought to be associated with a healthier and higher optimal organ perfusion with minimal disruption. Unfortunately, the effect and the cost of these treatments should both be considered; therefore, the current recommendations are still evolving. Recently, the American Heart Association (AHA) published the “Cardiac Arrest and Cardiopulmonary Resuscitation Outcome Reports” on September 16, 2019. The Utstein-style reporting templates were revised, and certain “core variables” were considered essential for the quality improvement programs [1]. Moreover, the European Resuscitation Council (ERC) also pointed out the importance of the CPR quality (ECPR, ECLS, and mechanical CPR) in their 2020 guidelines [2]. Several leading journals have also discussed how to increase the quality and outcome of CPR and the postcardiac arrest care (including strategic application of ECPR and hypothermia in treating pediatric or trauma patients). Applications of resuscitative endovascular balloon occlusion of the aorta (REBOA) for abdomen-pelvic hemorrhage and emergency vascular access for extracorporeal membrane oxygenation (ECMO) are also popular and timely [3–6]. In this special issue, we aim to focus on the field of emergency and critical care, particularly with a focus on the resuscitation of patients with a cardiac arrest and the care of patients during and after a cardiac arrest.

First, topics related to the use of videolight and conventional intubating stylet systems for emergency intubation were described in detail, and they were compared with other modes of intubation in COVID-19 setups. M-Y Wu et al. mentioned that digital devices can shorten intubation times, improve patient outcomes, and decrease the risk of infections among clinical staff, especially during the ongoing COVID-19 pandemic. In addition, M-F Wang et al. evaluated the CPR effectiveness that adolescents (12 years old) and adults could perform, and these are individuals who underwent the same courses for basic life support (BLS) and automated external defibrillators (AEDs). They found that the sixth-grade elementary students' performances in CPR and AED were similar to those of adults after completing the current 90-minute course. Therefore, they strongly advocated offering CPR and AED courses to 12-year-old children, and these courses should emphasize the importance of checking the breathing status of the patient.

The time and the process of transportation were key factors associated with patient outcomes. Puslecki et al. analyzed the safety of ECMO support during medical transportation, and this was a single-center study and literature review. They reviewed 2,647 ECMO transfers, most of which were transported by ground transportation (91.6%). The rate of adverse events ranged from 1% through 20%. Only 4 deaths occurred during transport (mortality 0.15%). Moreover, L-H Huang et al. evaluated the threshold of the ambulance response time for predicting the survival to hospital discharge for patients with an OHCA. After analyzing 6,742 adult OHCA cases, they found that the adjusted OR of the EMS response time for the survival to hospital discharge was 1.217 (for each minute shorter, CI: 1.140–1299) and 1.992 (<6.2 min, 95% CI: 1.496–2.653). In the case of an OHCA in public areas or with bystander CPR administration, the threshold was prolonged, and when medical personnel performed CPR, the optimal response time threshold was shortened.

Zalewski et al. designed a randomized simulation cross-study and assessed the impact of the defibrillation methods on the CPR quality. They concluded that the simulation-based analysis revealed that the use of adhesive electrodes during defibrillation instead of standard hard paddles may improve the quality of the CPR performed by a two-person emergency team. In addition, M-S Hsieh et al. focused on sepsis and the following outcomes. They reported the risks of gastrointestinal bleeding (GIB) and major adverse cardiovascular events (MACEs) in 220,082 sepsis patients. Both GIB and MACEs significantly increased the risk of ICU admission and the risk of patients receiving mechanical ventilation but did not increase the risk of hospital mortality, which was independently associated with both acute myocardial infarction (AMI) and intracranial hemorrhage (ICH). C-J Li et al. performed a multicenter retrospective cohort study analyzing the treatment effect of dopamine and norepinephrine on hypotension among OHCA patients who achieved return of spontaneous circulation (ROSC). They concluded that there were no significant differences in the 30-day survival and that there were favorable neurologic performance rates between the post-ROSC hypotension treatment groups (between patients who received dopamine and those who received norepinephrine).

Finally, this special issue highlights several articles that report improvements in the CPR training, quality control, resuscitation strategies, and sepsis outcomes. We believe that readers will obtain useful information from this article.

*Yan-Ren Lin* *Jacek Smereka* *Kee-Chong Ng* *John M. Ryan*

## References

[1] Nolan J. P., Berg R. A., Andersen L. W. (2019). Cardiac arrest and cardiopulmonary resuscitation outcome reports: update of the utstein resuscitation registry template for in-hospital cardiac arrest: a consensus report from a task force of the international liaison committee on resuscitation (American heart association, European resuscitation council, Australian and new Zealaouncil on resuscitation, heart and stroke foundation of Canada, InterAmerican heart foundation, resuscitation council of southern Africa, resuscitation council of asia). *Resuscitation*.

[2] Vadakkencherry Ramaswamy V., Abiramalatha T., Weiner G. M., Trevisanuto D. (2021). A comparative evaluation and appraisal of 2020 American heart association and 2021 European resuscitation council neonatal resuscitation guidelines. *Resuscitation*.

[3] Brenner M., Teeter W., Hoehn M. (2018). Use of resuscitative endovascular balloon occlusion of the aorta for proximal aortic control in patients with severe hemorrhage and arrest. *JAMA Surgery*.

[4] Abrams D., MacLaren G., Lorusso R. (2021). Extracorporeal cardiopulmonary resuscitation in adults: evidence and implications. *Intensive Care Medicine*.

[5] Lin Y.-R., Wu M.-H., Chen T.-Y. (2019). Time to epinephrine treatment is associated with the risk of mortality in children who achieve sustained ROSC after traumatic out-of-hospital cardiac arrest. *Critical Care*.

[6] Chang C. J., Liou T. H., Tsai W. T. (2020). Clinical and hematological predictors for return of spontaneous circulation in patients with out-of-hospital cardiac arrest. *Journal of acute medicine*.

